# Genetic disorders and pregnancy outcomes of non-immune hydrops fetalis in a tertiary referral center

**DOI:** 10.1186/s12920-023-01505-y

**Published:** 2023-04-20

**Authors:** Danhua Guo, Shuqiong He, Na Lin, Yifang Dai, Ying Li, Liangpu Xu, Xiaoqing Wu

**Affiliations:** Department of Medical Genetic Diagnosis and Therapy Center, Fujian Provincial Key Laboratory for Prenatal Diagnosis and Birth Defect, Fujian Maternity and Child Health Hospital College of Clinical Medicine for Obstetrics & Gynecology and Pediatrics, Fujian Medical University, Fuzhou City, Fujian Province People’s Republic of China

**Keywords:** Hydrops fetalis, Nonimmune, α- thalassemia, Chromosomal abnormality, Pregnancy outcome

## Abstract

**Objectives:**

Non-immune hydrops fetalis (NIHF) is a non-specific symptom associated with a wide range of disorders. The prognosis of NIHF depends on the underlying etiology. In this study, we investigated the incidence of chromosomal abnormalities and Bart’s hydrops fetalis in pregnancies associated with NIHF in South China.

**Methods:**

We conducted a retrospective review of NIHF pregnancies referred to the Fujian Provincial Maternity and Children’s Hospital between 2014 and 2018, excluding pregnancies with maternal alloimmunization. Routine karyotyping was performed on all 129 enrolled patients, and chromosomal microarray analysis was performed for 35 cases with a normal karyotype. In addition, α-thalassemia genotyping was performed to confirm the presence of Bart’s hydrops fetalis.

**Results:**

Chromosomal abnormalities were detected in 29.5% (38/129) of the cohort, including 37 cases with aneuploidy and one case with unbalanced structural rearrangement. Chromosomal microarray analysis performed on the 35 cases with a normal karyotype did not reveal any additional pathogenic variants. The proportions of chromosomal abnormalities declined with trimester progression, with frequencies of 65%, 30.1%, and 8.3% in the first, second, and third trimesters, respectively (p < 0.05). Bart’s hydrops fetalis was detected in 34.9% (45/129) of the cohort. Among the 46 (35.6%) cases with unknown etiology, 23 cases had other ultrasonic abnormalities characterized by poor outcomes, whereas seven cases with multiple cavity effusions that resolved or remitted prior to birth showed normal development during the 3–4 years of follow-up.

**Conclusions:**

In South China, Bart’s hydrops fetalis and chromosomal abnormalities are the most common genetic etiologies of NIHF. Generalized skin edema and accompanying ultrasonic abnormalities are predictive of adverse outcomes, highlighting the need for intensive monitoring and better pregnancy management of NIHF patients.

## Background

Hydrops fetalis (HF) is a fetal condition characterized by generalized skin edema or pathological fetal fluid collections in at least two body compartments, including ascites, pericardial effusion, pleural effusion, and skin edema (skin thickness > 5 mm) [1–3]. Polyhydramnios and placental thickening are also frequent sonographical findings associated with HF [4]. HF can be categorized as iso-immune HF (IHF) or non-immune HF (NIHF) based on causality, such as fetomaternal blood incompatibility. NIHF is a non-specific symptom that may occur in different trimesters and is associated with a wide range of disorders, including hematological and cardiovascular abnormalities, chromosomal anomalies, congenital infections, thoracic abnormalities, inborn errors of metabolism, lymphatic dysplasia, twin-to-twin transfusion syndrome, placental abnormalities, fetal tumors, and idiopathic causes [5]. According to the Society for Maternal-Fetal Medicine (SMFM), NIHF accounts for almost 90% of hydrops [6], with an incidence rate of 1 in 1700–3000 pregnancies worldwide [7–9]. NIHF affects 7.9 in 1000 pregnancies in Southern China [10]. Generally, NIHF presents a poor prognosis that varies from preterm birth to intrauterine fetal demise (IUFD), stillbirth, neonatal morbidity, and mortality [1, 11–13]. The prognosis of NIHF depends on the etiology and gestational age at the time of diagnosis and delivery [14]. Therefore, prenatal identification of the etiology of NIHF is essential for the evaluation of prognosis and recurrence risk, and for better pregnancy management. Accordingly, as per the guidelines in SMFM and our country, routine karyotyping and/or chromosomal microarray analysis (CMA) are generally offered to pregnancies with NIHF. In this view, we conducted a retrospective study to present our experiences with NIHF, regarding its genetic etiology and prognosis during pregnancy.

## Materials and methods

### Patients and samples

We retrospectively reviewed 360 singleton pregnancies that referred to Fujian provincial Prenatal Diagnostic Center due to NIHF, with or without other ultrasonic anomalies, including structural malformation, increased nuchal translucency(INT), nuchal cystic hygroma (NCH), arrhythmias, and fetal growth retardation (FGR), between January 2014 and December 2018. Cases with abnormal fluid collection involved at least two body compartments but without generalized skin edema were classified as multiple cavity effusions. Cases of multiple pregnancy, hydrops resulting from maternal alloimmunization, congenital infection, and cases without genetic analysis were excluded from the study. As a result, 129 cases that underwent invasive prenatal genetic testing were enrolled in this study, including 50 cases (38.7%) of isolated NIHF and 79 cases (61.3%) of NIHF with at least one other ultrasonic anomaly. The enrolled cases were categorized into three groups based on the gestational age (GA) at which NIHF was initially diagnosed: the first trimester group (~ 13^+ 6^ W) included 20 cases, the second trimester group (14 ~ 27^+ 6^ W) included 73 cases and the third trimester (≥ 28 W) group included 36 cases. The gestational age when NIHF was initially detected ranged from 11 to 38 weeks, with a median of 23 weeks. The maternal age of NIHF ranged from 18 to 45 years, with a median of 29 years. The demographics characters are presented in Table 1.

**Table 1:** The demographics characters of the 129 pregnancies of NIHF

	Value
Maternal age (Years), (Range, Median, Mean ± SD)	18–45, 29, 29.8 ± 5.4
Trimester at NIHF initially detected (Range, Median, Mean ± SD)	11–38, 23, 22.6 ± 7.2
First trimester (n, %)	20, 15.5
Second trimester (n, %)	73, 56.6
Third trimester (n, %)	36, 27.9
Specimens	
CV (n, %)	20,15.5
AF (n, %)	65,50.4
CB (n, %)	44,34.1

CV, chorionic villi; AF, amniotic fluid; UCB, umbilical cord blood

Due to the high incidence of α-thalassemia in Southern China, all parents of NIHF underwent blood routine examination and hemoglobin electrophoresis screening. Thalassemia gene analysis was done if necessary. If both parents were carriers of the same type of thalassemia, a thalassemia gene test was conducted on the invasive prenatal samples concurrently.

The specimens included 20 cases of chorionic villi (CV) sampled collected between 11–13+ 6 gestational weeks, 65 cases of amniotic fluid (AF) obtained between 18 and 24 gestational weeks, and 44 cases of umbilical cord blood (UCB) obtained between 25 and 35 gestational weeks. Routine karyotyping was performed in all the 129 cases, of which, 35 cases underwent concurrent CMA, and 45 cases underwent simultaneous testing for thalassemia genes and karyotyping.

### Karyotype analysis

Prenatal samples cell culture and G-banded karyotyping were performed according to the standard protocols in our laboratory. Karyotyping analysis was at a resolution level of 320–500 bands.

### Chromosomal microarray analysis

Karyotyping was performed as previously published [15]. Whole genomic DNA was extracted from chorionic villi, amniotic fluid or umbilical cord blood using a Qiagen kit (Qiagen GmbH, Hilden, Germany) following the manufacturer’s instructions. Chromosomal microarray analysis was performed using Affymetrix CytoScan 750 K array (Affymetrix Inc., Santa Clara, CA, UA), including 200,000 probes for single nucleotide polymorphisms and 550,000 probes for copy number variations (CNVs). The resulting scan data were interpreted using Chromosome Analysis Suite software (Affymetrix) and human genome version GRCh37 (hg19). All detected CNVs were compared with in-house and national public CNV databases, including the Database of Chromosome Imbalance and Phenotype in Humans Using Ensemble Resources (DECIPHER), Database of Genomic Variants (DGV), International Standards for Cytogenomic Arrays Consortium, and Online Mendelian Inheritance in Man (OMIM).

The CNVs were divided into five groups according to the American College of Medical Genetics (ACMG) [16]: pathogenic, likely pathogenic, variants of uncertain significance (VOUS), likely benign and benign. Pathogenic or likely pathogenic CNVs were considered as clinically significant findings. Parental CMA was performed to determine the inheritance of CNVs.

### Thalassemia genotyping

As previously described in our publication [17], genomic DNA was extracted from the umbilical cord blood, amniotic fluid samples or chorionic villi samples using the genomic DNA isolation kit protocol (Qiagen; Hilden, Germany). Deletional α-thalassemia were detected by Gap-PCR, and point mutations of α-thalassemia and β-thalassemia detection were detected by reverse dot-blot hybridization (RDB) using the thalassemia gene detection kit (Shenzhen Yishengtang Biological Products Co., Ltd.; Shenzhen, China) [18, 19]. α^0^-thalassemia is defined when both of the linked α globin genes are deleted or reduced in activity by mutation, and α^+^- thalassemia is defined when only one linked α globin gene is involved. The compound heterozygous states for α^+^-thalassemia and α^0^-thalassemia is termed as hemoglobin H disease; deletion or inactivation of all four α-globin genes leads to a lethal condition named hemoglobin Bart’s hydrops fetalis [20].

### Clinical follow-up

Follow-up information was available for 124 cases (96.1%, 124/129). Pregnancy outcomes were categorized into termination of pregnancy (TOP), intrauterine fetal death (IUFD), early neonatal death (END), and live births with normal growth and development. Clinical follow-up was conducted by reviewing medical records and conducting telephone calls.

### Statistical analyses

Data were analyzed using SPSS software version 22.0 (IBM, Armonk, NY). Statistical comparisons were performed using the chi-square test or Fishers exact test. Statistical significance was defined using a P value of < 0.05.

## Results

The results showed that 38 out of 129 cases (29.5%) had chromosomal abnormalities identified through routine karyotyping. Among these cases, 34 had numerical abnormalities, including 16 cases of trisomy 21 (T21), 12 of 45,X, 6 of trisomy 18 (T18), 1 of mosaic trisomy 20 (T20), 1 of mosaic trisomy 13 (T13), 1 of 47,XXY, and one with unbalanced structural abnormalities: 46,XN,der (1)t(1;15) (q44;q14)mat, -15. More details can be found in Table 2.

**Table 2:** Details in pregnancies of NIHF with abnormal chromosomes

Case NO.	GA at diagnosis (weeks)	Maternal age (years)	Ultrasound findings	Karyotype	Outcomes
1	11	38	CVM; GSE	45, X	TOP
2	12	26	GSE, gastroschisis	47, XY, + 20[15]/46, XY[30]	TOP
3	12	43	GSE, INT	47, XY, + 21	TOP
4	12	45	GSE	47, XY, + 18	TOP
5	12	31	GSE, INT	47, XX, + 21	TOP
6	13	30	GSE, INT	45, X	TOP
7	13	32	GSE, NCH, bilateral pleural effusion, CVM	46,XY,der(1)t(1;15)(q44,q14)mat ，− 15	TOP
8	13	37	GSE, INT	47, XX, + 21.	TOP
9	13	29	GSE, NCH	45, X	TOP
10	13	26	GSE, INT, CVM, holoprosencephaly, omphalocele	47, XX + 13 [12]/46, XX [28]	TOP
11	13	24	GSE, bilateral pleural effusion, CVM	45, X	TOP
12	13	29	GSE, NCH	45, X	TOP
13	13	41	GSE, NCH, INT	47, XY, + 21	TOP
14	14	28	GSE, NCH	45, X	TOP
15	14	36	GSE, NCH	47, XY, + 18	TOP
16	14	30	GSE, pleural effusion, CVM	47, XY, + 21	TOP
17	14	41	GSE	47, XY, + 21	TOP
18	15	25	GSE, NCH, CVM, omphalocele, skeletal malformation	47, XY, + 18	TOP
19	17	34	GSE, NCH	47, XY, + 18	TOP
20	17	24	GSE, NCH, talipes	47, XX, + 18	IUFD
21	18	23	GSE, pleural effusion, ascites, pericardial effusion	47, XY, + 21	TOP
22	18	41	GSE, NCH, CVM	45, X	TOP
23	18	29	GSE, CVM, pleural effusion, ascites	47, XY, + 21	IUFD
24	19	31	GSE, NCH, pleural effusion, ascites	45, X	TOP
25	19	23	NCH, bilateral pleural effusion	47, XY, + 21	TOP
26	19	29	GSE, NCH, CVM, placental thickening	45, X	TOP
27	20	33	GSE, NCH, CVM, FGR	45, X	TOP
28	22	37	NCH, CVM, pericardial effusion	47, XX, + 21	TOP
29	23	35	bilateral pleural effusion, ascites	45, X	TOP
30	23	28	pleural effusion, ascites, FGR, cardiomegaly	45, X	TOP
31	23	42	GSE, bilateral pleural effusion, ascites	47, XY, + 21	IUFD
32	24	28	GSE, bilateral pleural effusion	47, XXY	IUFD
33	24	20	pleural effusion, ascites, CVM	47, XY, + 21	TOP
34	26	34	pericardial effusion, ascites, FGR, placental thickening, CVM	47, XY ,+21	TOP
35	27	28	pleural effusion, ascites, pericardial effusion, CVM, polyhydramnios	47, XY, + 18	TOP
36	28	41	GSE, bilateral pleural effusion, polyhydramnios, local skin edema	47, XY, + 21	TOP
37	30	41	pleural effusion, ascites, polyhydramnios, CVM	47, XX, + 21	TOP
38	32	28	bilateral pleural effusion, polyhydramnios, CVM	47, XY, + 21	TOP

Abbreviations: TOP, termination of pregnancy; IUFD, intrauterine fetal demise; INT, increased nuchal translucency;

FGR, fetal growth retardation; GSE, generalized skin edema; NCH, nuchal cystic hygroma; CVM, cardiovascular malformation

Among the 129 cases of NIHF, 20 cases occurred in the first trimester, 73 cases occurred in second trimester, and 36 cases occurred in third trimester. As shown in Table 3, the rates of chromosomal abnormalities in the three trimesters were 65%, 30.1%, and 8.3%, respectively (p<0.05 ). Excluding the Bart’s hydrops fetalis, NIHF accompanied by other ultrasonic abnormalities had a higher rate of chromosomal abnormalities (59.3%, 32/54) than fetuses without other ultrasonic abnormalities (20.0%, 6/30) (p < 0.05).

**Table 3:** Distribution of chromosomal abnormalities and Bart’s hydrops in different trimesters

Trimesters	Abnormal Karyotype (n, %)	Fetuses with normal karyotype			Total
		Non-Bart’s Hydrops	Bart’s Hydrops	Total	
First	13, 65.0%	7, 35.0%	0, 0.0%	7, 35.0%	20
Second	22, 30.1%	19, 26.0%	32, 43.8%	51, 69.9%	73
Third	3, 8.3%	20, 55.6%	13, 36.1%	33, 91.7%	36

Among the 35 cases underwent concurrent CMA and routine karyotyping, CMA detected one additional aberration compared to routine karyotyping. The affected fetus showed a 3.4 Mb duplication at the region of 3p14.1p13[arr[hg19] 3p14.1p13(66,483,797 − 69,908,461)x3]. This variant was interpreted as a benign CNV because it was inherited from the mother, who had a normal phenotype.

### Thalassemia genotyping

In our cohort, 45 couples both parents were confirmed as α^0^-thalassemia carriers, and their NIHF fetus were diagnosed with Bart’s hydrops. For another two couples, one of the partners was confirmed as α^+^-thalassemia, the etiology of their NIHF fetus remained unknown. Bart’s hydrops fetalis accounted for 34.9% (45/129) of NIHF cases in our study, particularly in second and third trimesters, representing 43.8% (32/73) and 36.1% (13/36) respectively. No Bart’s hydrops related HF cases occurred in the first trimester. Details are summarized in Table 4.

**Table 4:** Details of the 45 cases with Bart’s hydrops

Case NO.	GA at diagnosis (weeks)	Maternal age	Ultrasound Imaging	Karyotype		outcomes
39	17	25	ascites, pericardial effusion, cardiomegaly	46, XX		TOP
40	17	33	ascites, pericardial effusion, cardiomegaly, placental thickening, MCA PSV >1.55MoM	46, XX		TOP
41	18	31	ascites, pericardial effusion, placental thickening, MCA PSV >1.55MoM	46, XX		TOP
42	19	29	ascites, pericardial effusion, placental thickening, cardiomegaly, MCA PSV >1.55MoM	46, XY		TOP
43	19	25	ascites, pericardial effusion, MCA PSV >1.55MoM	46, XX		TOP
44	19	28	ascites, pericardial effusion, placental thickening ,cardiomegaly ,MCA PSV >1.55MoM	46, XY		TOP
45	19	25	ascites, pleural effusion, placental thickening ,cardiomegaly ,MCA PSV >1.55MoM	46, XX		TOP
46	19	29	ascites, pericardial effusion, placental thickening ,cardiomegaly ,MCA PSV >1.55MoM	46, XX		TOP
47	20	18	ascites, pericardial effusion, placental thickening ,cardiomegaly ,MCA PSV >1.55MoM	46, XY		TOP
48	21	26	ascites, pericardial effusion	46, XX		TOP
49	21	32	ascites, pleural effusion	46, XX		TOP
50	21	29	generalized skin edema, MCA PSV >1.55MoM	46, XX		TOP
51	22	26	ascites, pericardial effusion, placental thickening, polyhydramnios	46, XX		TOP
52	22	30	ascites, pericardial effusion, placental thickening ,cardiomegaly ,MCA PSV >1.55MoM	46, XY		TOP
53	22	26	ascites, pericardial effusion ,cardiomegaly ,MCA PSV >1.55MoM	46, XX		TOP
54	23	26	ascites, pericardial effusion	46, XX		TOP
55	23	27	ascites, pericardial effusion, placental thickening, cardiomegaly	46, XY		TOP
56	23	31	generalized skin edema, ascites, pericardial effusion, placental thickening ,cardiomegaly ,MCA PSV >1.55MoM	46, XY		TOP
57	23	32	ascites, pericardial effusion, placental thickening ,cardiomegaly ,MCA PSV >1.55MoM	46, XY		TOP
58	23	38	pleural effusion, pericardial effusion, placental thickening ,cardiomegaly ,MCA PSV >1.55MoM	46, XX		TOP. Maternal mirror syndrome
59	24	26	ascites, pericardial effusion, cardiomegaly ,MCA PSV >1.55MoM	46, XX		TOP
60	24	28	ascites, pericardial effusion, cardiomegaly	46, XY		TOP
61	24	30	ascites, pericardial effusion, cardiomegaly, MCA PSV >1.55MoM	46, XY		TOP
62	24	28	ascites, pericardial effusion, FGR, cardiomegaly	46, XY		TOP
63	25	30	ascites, pericardial effusion, placental thickening, cardiomegaly	46, XX		TOP
64	25	28	ascites, pericardial effusion, FGR, cardiomegaly	46, XX		TOP
65	25	27	ascites, pericardial effusion, FGR, cardiomegaly	46, XY		TOP
66	25	24	ascites, pericardial effusion, cardiomegaly, MCA PSV >1.55MoM	46, XY		TOP
67	25	26	ascites, pericardial effusion, cardiomegaly	46, XY		TOP
68	26	28	ascites, pericardial effusion, placental thickening, cardiomegaly	46, XX		TOP
69	27	22	ascites, pericardial effusion, placental thickening, cardiomegaly	46, XY		TOP
70	27	23	ascites, pericardial effusion, cardiomegaly	46, XX		TOP
71	28	32	ascites, pericardial effusion, placental thickening, cardiomegaly	46, XY		TOP
72	29	28	ascites, pericardial effusion	46, XY		TOP
73	29	34	ascites, pericardial effusion, placental thickening, cardiomegaly	46, XX		TOP
74	29	31	ascites, pleural effusion, placental thickening, cardiomegaly, FGR	46, XX		TOP
75	30	30	ascites, pericardial effusion, cardiomegaly, polyhydramnios, FGR	46, XY		TOP
76	31	29	ascites, pericardial effusion, placental thickening, cardiomegaly	46, XX		Premature delivery, END
77	31	35	ascites, pericardial effusion, placental thickening, cardiomegaly, local skin edema	46, XY		Premature delivery, END
78	31	30	ascites, pericardial effusion, placental thickening, cardiomegaly	46, XX		TOP
79	31	24	ascites, pericardial effusion, placental thickening, cardiomegaly, FGR	46, XX		IUFD
80	32	20	ascites, pericardial effusion, local skin edema, placental thickening, polyhydramnios, FGR	46, XY		Maternal mirror syndrome. Premature delivery, END
81	32	26	generalized skin edema, ascites, FGR	46, XY		TOP
82	32	18	ascites, bilateral pleural effusion, placental thickening ,cardiomegaly ,MCA PSV >1.55MoM	46, XX		Maternal mirror syndrome. Premature delivery, END
83	33	28	ascites, pericardial effusion, placental thickening, cardiomegaly	46, XY		TOP

Abbreviations: TOP, termination of pregnancy; IUFD, intrauterine fetal demise; END, early neonatal death; FGR, fetal growth retardation; NT, nuchal translucency; MCV PSV, middle cerebral artery peak systolic velocity; MOM, multiples of the median

### Pregnancy outcome

Follow-up information was available for 124 (96.1%) cases, including 97 (78.2%) cases of termination, 13 (10.5%) cases of intrauterine fetal demise (IUFD), 7 (5.6%) cases of early neonatal deaths (END), and 7 (5.7%) cases with normal development at 3–4 years of follow-up. All NIHF cases with genetic abnormalities were associated with termination of pregnancy (TOP) or other adverse outcomes. Details are summarized in Table 5.

**Table 5:** Outcomes for 124 pregnancies with NIHF

	Total			Others			
		Abnormal karyotype (n, %)	Bart’s hydrops	Generalized skin edema and other*	Generalized skin edema and other*	Generalized skin edema and other*	Multiple cavity effusions only
TOP	97,78.2%	34,89.5%	40,88.9%	10, 71.4%	8, 88.9%	2, 33.3%	3, 25.0%
IUFD	13,10.5	4,10.5%	1,2.2%	4, 28.6%	1, 11.1%	2, 33.3%	1, 8.3%
END	7,5.6%	0 ,0.0%	4,8.9%	0, 0.0%	0, 0.0%	2, 33.4%	1, 8.4%
Live birth with normal development	7,5.7%	0,0.0%	0,0.0%	0, 0.0%	0, 0.0%	0, 0.0%	7, 58.3%
Total	124,100%	38 ,100%	45, 100%	14, 100%	9, 100.0%	6, 100%	12, 100.0%

Abbreviations: multiple cavity effusions, ascites in at least two body compartments but without generalized skin edema

*: other ultrasonic abnormalities, including structural malformation, increased nuchal translucency(INT), nuchal cystic hygroma(NCH), arrhythmias, fetal growth retardation (FGR)

Among the 41 cases with unknown genetic results, the survival rate was 17.1%. All 23 cases accompanied by other ultrasonic abnormalities resulted in poor outcomes. Of the 18 cases without other ultrasonic abnormalities, 7 cases showed normal development during 3 to 4 years of follow-up (all the 7 cases showed multiple cavity effusions, of which 5 cases resolved prior to birth, and 2 cases showed remission prior to birth). However, the remaining 11 cases with persisting or progressive hydrops had poor outcomes.

A total of 18 cases with unknown etiology chose to continue their pregnancies at the initial diagnosis. Of the 8 fetuses with generalized skin edema, all had adverse outcomes, While the remaining 10 fetuses exhibited multiple cavity effusions, of which 3 had adverse outcomes and 7 had favorable outcomes. (*χ²*=9.164, P = 0.004).

Details of NIHF with unknown genetic abnormalities are provided in Table 6.

**Table 6:** Details of NIHF with unknown etiology

Case NO.	GA at diagnosis (weeks)	Maternal age	Ultrasound Imaging	Karyotype	CMA Results	Outcomes
84	12	30	GSE, NCH	46, XX	N	TOP
85	12	35	GSE, INT	46, XX	N	TOP
86	12	31	GSE, gastroschisis	46, XY	N	TOP
87	12	29	GSE	46, XX	N	IUFD
88	12	28	GSE, INT, CVM	46, XX	NA	TOP
89	13	29	GSE, INT, cleft lip and palate	46, XX	NA	TOP
90	13	35	GSE, INT, bilateral pleural effusion	46, XX	N	PA, IUFD
91	14	27	ascites, pericardial effusion, CVM,	46, XY	N	TOP
92	15	19	GSE	46, XX	N	PA, IUFD
93	15	25	GSE, NCH	46, XY	N	TOP
94	21	35	GSE, NCH, CVM, placental thickening	46, XX	N	TOP
95	22	32	GSE, pleural effusion, ascites, cleft lip and palate, arrhythmias	46, XX	N	TOP
96	23	26	ascites, pericardial effusion, FGR, placental thickening, cardiomegaly, pulmonary dysplasia	46, XY	N	TOP
97	23	28	ascites, pericardial effusion, cardiomegaly, sacrococcygeal region teratoma, polyhydramnios	46, XY	N	TOP
98	23	30	bilateral pleural effusion, CVM	46, XX	N	TOP
99	23	26	bilateral pleural effusion	46, XX	N	RA, term(38W), CS, drainage, ND at 4-year follow-up
100	23	33	GSE, NCH, CVM	46, XX	NA	TOP
101	24	32	GSE, FGR	46, XY	N	PA, IUFD
102	24	34	ascites, pericardial effusion, cardiomegaly, polyhydramnios	46, XX	N	PA, IUFD
103	24	29	pericardial effusion, local skin edema	46, XY	N	RA ,Term(39W),CS, ascites absorpted , ND at 4-year follow-up
104	25	36	GSE, NCH	46, XY	N	PA, IUFD
105	25	26	ascites, pleural effusion	46, XX	N	TOP
106	26	25	GSE, ascites, pleural effusion, tachyarrhythmias	46, XY	N	PA, IUFD
107	27	26	GSE, polyhydramnios, cardiomegaly	46, XY	N	TOP
108	27	36	pleural effusion, local skin edema, NCH	46, XX	N	TOP
109	28	30	ascites, pericardial effusion, CVM, placental thickening, local skin edema	46, XY	N	TOP
110	28	36	ascites, bilateral pleural effusion	46, XY	N	PA, IUFD
111	28	28	GSE	46, XY	N	TOP
112	28	27	ascites, local skin edema	46, XX	NA	TOP
113	31	32	bilateral pleural effusion, local skin edema	46, XY	N	RA ,Term(41+4w),Natural delivery,edema absorpted , ND at 3-year follow up
114	31	24	ascites, bilateral pleural effusion, CVM	46, XX	N	TOP
115	31	33	ascites, pleural effusion	46, XY	N	TOP
116	32	36	GSE	46, XX	N	PA, Preterm(35+3w),Natural delivery, END
117	33	28	ascites, local skin edema	46, XY	NA	RA ,Term(37 W),Natural delivery, ascites absorpted , ND at 4-year follow up
118	33	26	ascites, pericardial effusion, cardiomegaly, polyhydramnios	46, XY	N	TOP
119	33	38	bilateral pleural effusion, polyhydramnios	46, XX	NA	PA, preterm(34W), Natural delivery, END
120	35	27	ascites, pleural effusion	46, XY	N	RA, preterm(36W), CS, drainage, ND at 4-year follow up
121	36	27	GSE	46, XY	N	TOP
122	36	24	GSE	46, XY	NA	PA, preterm(36W), Natural delivery, END
123	36	32	bilateral pleural effusion, polyhydramnios	46, XY	N	RA ,preterm(36+5W),CS,drainage, ND at 3-year follow up
124	38	19	pericardial effusion, local skin edema	46, XX	NA	RA, Term(39+1W),CS, ascites absorbed , ND at 3-year follow up
125	24	23	ascites, pericardial effusion, placental thickening ,cardiomegaly ,MCA PSV >1.55MoM	46, XX	N	Lost to follow up
126	28	39	bilateral pleural effusion, polyhydramnios, CVM	46, XX	N	Lost to follow up
127	28	33	ascites, pericardial effusion, placental thickening, cardiomegaly	46, XY	NA	Lost to follow up
128	35	34	bilateral pleural effusion	46, XY	N	Lost to follow up
129	37	39	GSE, ascites, pleural effusion, polyhydramnios, CVM	46, XY	NA	Lost to follow up

Abbreviations: GA, gestational age; TOP, termination of pregnancy; IUFD, intrauterine fetal demise; INT, increased nuchal translucency;

FGR, fetal growth retardation

GSE, generalized skin edema; NCH, nuchal cystic hygroma; CVM, cardiovascular malformation

CS, cesarean section

ND, normal development

RA, remission antenatal

PA, progression antenatal

N, normal

NA, not available

## Discussion

In this study, we evaluated the genetic etiology of 129 pregnant women with NIHF, based on the results of routine karyotyping, thalassemia genotyping, and CMA analysis. Genetic abnormalities were identified in 64.3% of the cohort, with hematological disease and chromosomal abnormalities constituting 34.9% and 29.5% of the NIHF pregnancies, respectively.

Although chromosomal abnormalities are frequently observed in NIHF [1], the precise mechanisms underlying this association remain unclear. In our study, chromosomal abnormalities were identified as the second most prominent cause of NIHF. Consistent with previous reports [1, 21, 22], aneuploidy, particularly that associated with Down syndrome, Turner syndrome, and Edwards syndrome, was the most common chromosomal abnormality. In pregnancies which NIHF occurred during the first trimester, we identified chromosomal abnormalities in 65% of cases, which is comparable to the rate reported by Jenewein et al. [21] (61%, 25/41), and Filomena et al. [23](68.9%, 44/63), but considerably higher than the rate reported by Ramkrishna et al. [24]( 19.2%, 20/104). For the second trimester, Heinonen et al. [7] reported that chromosomal abnormalities accounted for 44.8% (26/58) of NIHF cases, which is somewhat higher than the 30.1% observed in our study. We believe that this discrepancy may be due to variations in the number of samples analyzed. To our knowledge, this is the first study in China to investigate the prevalence of NIHF-associated chromosomal abnormalities across all trimesters. We found that the proportion of detected chromosomal abnormalities decreased as pregnancy progressed. Furthermore, we found that an earlier onset of NIHF is associated with a higher incidence of chromosomal abnormalities. Specifically, 78.9% (30/38) of the chromosomal abnormalities were found to be associated with nuchal cystic hygroma and/or structural anomalies. Possible causes of early onset NIHF include concurrent multi-system abnormalities, such as cardiac anomalies, lymphatic dysplasia, and abnormal myelopoiesis. These abnormalities can lead to early-onset edema, although not all may be discovered on early ultrasonic scans due to the small gestational age and limited resolution.

In this retrospective study, all the detected chromosome abnormalities were diagnosed through routine karyotyping. Aneuploidy was found in 97.4% (37/38) of the cases, while CMA analysis of the 35 cases with normal karyotypes did not reveal any additional pathogenic variants. This finding is consistent with previous studies [24–26]. However, in contrast to our findings, Deng et al. achieved an additional detection rate of 4.2% (3/72) in NIHF using CMA [22]. The discrepancy between the studies could be attributed to the small number of samples assessed in our study. Therefore, a larger sample size is needed to adequately evaluate the application of CMA in NIHF.

Hematological abnormalities are a well-established etiology of NIHF, with α-thalassemia being the most common monogenic disease worldwide [20]. Thalassemia typically presents with minimal, microcytic, and hypochromic anemia, although most α^0^-thalassemia and α^+^- thalassemia are clinically asymptomatic. Hemoglobin H disease can lead to thalassemia intermedia, which does not require transfusions or sporadic transfusions, whereas Bart’s hydrops is a lethal condition that generally results in IUFD or END [20]. In China, α-thalassemia is highly prevalent in Fujian Province, with a prevalence rate of 4.84%, of which 67.4% are α^0^- thalassemia [14]. In this study, Bart’s hydrops was identified as the most common cause of NIHF (34.9%) and was primarily detected in pregnancies where NIHF occurred during the second and third trimesters. Moreover, in all 45 cases, Bart’s hydrops fetalis led to poor pregnancy outcomes. It is worth noting that the proportion of NIHF cases associated with Bart’s hydrops (34.9%) in this study is higher than that reported earlier in Guangzhou (28.4%) [10]. We suspect the discrepancy could be due to the prevalent screening and diagnosis of thalassemia in Guangzhou, where screening is initiated at an earlier stage. Couples at risk of inheriting this monogenic blood disorder can take reproductive measures to reduce the likelihood of NIHF caused by Bart’s hydrops, such as pre-implantation genetic diagnosis, early prenatal diagnosis, or gamete donation.

The SMFM recommends karyotyping, CMA, fetal echocardiography, and DNA analysis for the diagnosis of NIHF, as well as for single-gene etiologies. However, even when using a combined approach based on karyotyping and CMA, along with a hydrops gene panel and targeted genetic sequencing, genetic etiology was detected in only 25% of the assessed NIHF cases [27]. Additionally, single-gene disorders are responsible for the development of NIHF. Quinn et al. categorized 131 genes with strong evidence of association with NIHF and 46 genes with emerging evidence, including genes associated with cardiac, hematological, and metabolic disorders [28]. Due to the limited resolution of karyotyping and CMA, the etiology of 35.7% of the NIHF cases in the present study remains undetermined. In such cases, next-generation sequencing could be considered for screening NIHF cases characterized by a normal karyotype.

The prognosis of NIHF depends on the underlying etiology, genetic abnormalities, and structural anomalies associated with unfavorable outcomes [1, 29, 30]. In the present study, all cases of Bart’s hydrops and chromosomal abnormalities resulted in poor outcomes, and pregnancy outcomes in patients with NIHF differed according to ultrasonic presentation. Reischer et al. have reported that adverse outcomes are more common in cases where more compartments are affected [31]. In the present study, we found that outcomes were less favorable for fetuses with generalized skin edema than for those with multiple cavity effusions, suggesting generalized skin edema could be considered a predictor of poor outcome. Additionally, antenatally resolved HF has been shown to have a more favorable prognosis than that persists[29]. In our study, we found that seven cases with prenatal hydrops resolution or remission had favorable outcomes, whereas the outcomes for all 11 fetuses with persisting or progressive hydrops were generally poor. These findings suggest intensive monitoring should be provided for idiopathic NIHF to obtain a better evaluation of prognosis.

Nevertheless, this study has certain limitations, although it provides valuable insights into the genetic etiology of NIHF. Notably, not all NIHF patients underwent CMA analysis. Additionally, the retrospective descriptive design lacked a control group, and the small sample size limited our ability to analyze different outcomes among subgroups with continued pregnancy.

Overall, the findings of this study indicate that Bart’s hydrops and chromosomal abnormalities are the most common genetic etiologies of NIHF in Fujian Province. We also established that the incidence of chromosomal abnormalities declines with trimester progression. However, there was limited evidence to suggest that CMA can improve the rate of detecting chromosomal abnormalities in NIHF fetuses. Therefore, intensive monitoring of NIHF patients is necessary to improve pregnancy management.

## Data Availability

All data generated or analyzed during this study are included in this published article and its supplementary information files.
